# The BET bromodomain inhibitor, JQ1, facilitates c-FLIP degradation and enhances TRAIL-induced apoptosis independent of BRD4 and c-Myc inhibition

**DOI:** 10.18632/oncotarget.5785

**Published:** 2015-09-22

**Authors:** Weilong Yao, Ping Yue, Fadlo R. Khuri, Shi-Yong Sun

**Affiliations:** ^1^ Department of Respiration, Xiangya Hospital and Xiangya School of Medicine, Central South University, Changsha, Hunan, PR China; ^2^ Department of Hematology and Medical Oncology, Winship Cancer Institute, Emory University School of Medicine, Atlanta, Georgia, USA

**Keywords:** JQ1, c-FLIP, TRAIL, apoptosis, BRD4, c-Myc

## Abstract

Inhibition of BET bromodomains (BRDs) has emerged as a promising cancer therapeutic strategy. Accordingly, inhibitors of BRDs such as JQ1 have been actively developed and some have reached clinical testing. However, the mechanisms by which this group of inhibitors exerts their anticancer activity, including induction of apoptosis, have not been fully elucidated. This report reveals a previously uncovered activity of JQ1 in inducing c-FLIP degradation and enhancing TRAIL-induced apoptosis. JQ1 potently decreased c-FLIP (both long and short forms) levels in multiple cancer cell lines without apparently increasing the expression of DR5 and DR4. Consequently, JQ1, when combined with TRAIL, synergistically induced apoptosis; this enhanced apoptosis-inducing activity could be abolished by enforced expression of ectopic FLIP_L_ or FLIP_S_. Hence it appears that JQ1 decreases c-FLIP levels, resulting in enhancement of TRAIL-induced apoptosis. Inhibition of proteasome with MG132 prevented JQ1-induced c-FLIP reduction. Moreover, JQ1 decreased c-FLIP stability. Therefore, JQ1 apparently decreases c-FLIP levels through facilitating its proteasomal degradation. Genetic inhibition of either BRD4 or c-Myc by knocking down their expression failed to mimic JQ1 in decreasing c-FLIP and enhancing TRAIL-induced apoptosis, suggesting that JQ1 induces c-FLIP degradation and enhances TRAIL-induced apoptosis independent of BRD4 or c-Myc inhibition. In summary, our findings in this study highlights a novel biological function of JQ1 in modulating apoptosis and warrant further study of the potential treatment of cancer with the JQ1 and TRAIL combination.

## INTRODUCTION

The bromodomain (BRD) and extra-terminal domain (BET) family is comprised of four proteins: BRD2, BRD3, BRD4, and BRDT, which perform diverse roles in regulating gene transcription. These BET family proteins have been identified in oncogenic rearrangements, generating highly oncogenic fusion proteins, and in regulating transcription of several oncogenes, such as c-Myc and Bcl-2. Therefore they play key roles in oncogenesis of certain types of cancer and accordingly, targeting BET proteins has emerged as a promising cancer therapeutic strategy [1, 2].

**Figure 1:**
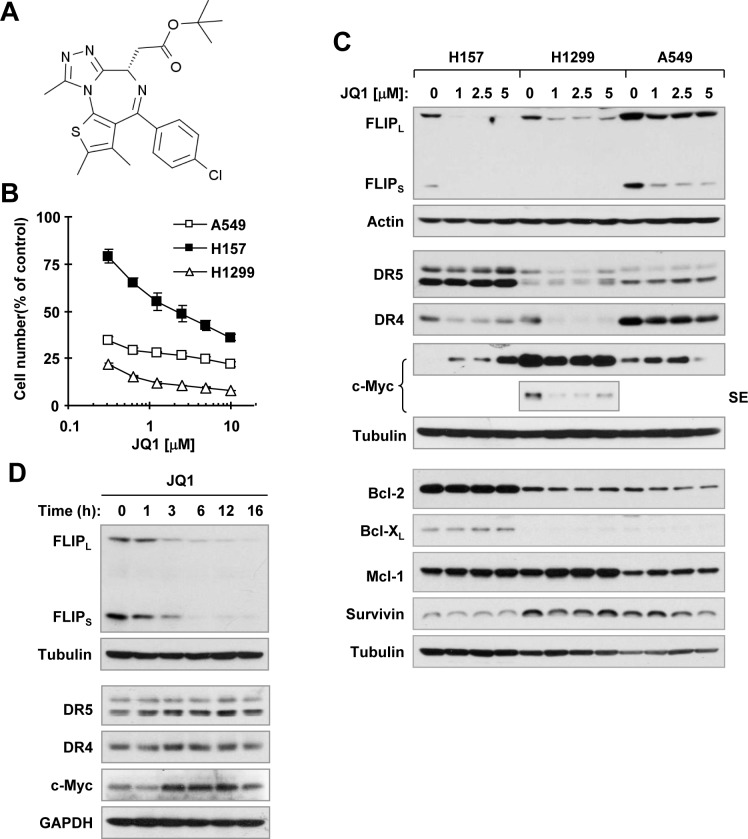
JQ1 (A) decreases the survival of cancer cells (B) and decreases c-FLIP levels (C and D) **A**, Chemical structure of JQ1. **B**, The indicated cancer cell lines were treated with different concentrations of JQ1 for 3 days and then subjected to estimation of cell number with the SRB assay. The data are means ± SDs of four replicate determinations. **C** and **D**, The indicated cell lines were treated with the given concentrations of JQ1 for 12 h **(C)** or 3 μM for the different times as indicated **(D)** and then harvested for preparation of whole-cell protein lysates and subsequent Western blot analysis. SE, short exposure.

In the past few years, several small molecule inhibitors that target BET family proteins, particularly BRD4, have been developed. These inhibitors have been actively used either as therapeutic agents or as research tools in many preclinical studies and some of them have advanced to testing in clinical trials [1, 2]. JQ1, a triazolothienodiazepine (Figure 1A), is the first BET BRD inhibitor developed for cancer therapy [3, 4] and hence has been widely used. One initial putative mechanism by which JQ1 and other BET BRD inhibitors exert their anticancer activity is through suppression of c-Myc expression [3, 5]. However, other mechanisms involving regulation of a different set of cancer-relevant genes independent of c-Myc have also been suggested [6, 7]. By far, the majority of studies have shown that the therapeutic effect of BET BRD inhibitors is attributed to targeting BRD4, rather than the other BET proteins [2].

JQ1 and other BET BRD inhibitors have been reported to induce apoptosis in some types of cancer cells including lung cancer cells, contributing to their anticancer activity [8-14]. However, the underlying mechanisms of apoptosis induction are largely unclear other than Bim involvement [10, 12]. In addition to single agent activity, these inhibitors also act synergistically with other cancer therapeutic agents such as HDAC inhibitors [15, 16], mTOR inhibitors [13, 17, 18], and PI3K inhibitors [19] in inducing apoptosis and inhibiting the growth of cancer cells and tumors. Further evaluation of these combinations in the clinic is thus warranted even though continuous investigation of their biological actions is still needed.

Tumor necrosis factor-related apoptosis-inducing ligand (TRAIL; also called APO-2L) is a well-known death ligand or cytokine that initiates apoptosis upon ligation with two death receptors named death receptor 4 (DR4) and 5 (DR5). TRAIL preferentially induces apoptosis in transformed or malignant cells, but not in most normal cells, and thus is a tumor-selective apoptosis-inducing cytokine with cancer therapeutic potential [20, 21]. Recombinant human TRAIL is currently being tested in clinical trials as a potential cancer therapeutic agent [21-23]. Unfortunately cancer cells exhibit varied sensitivity to TRAIL and a substantial proportion of cancer cell lines are intrinsically insensitive to TRAIL [20]. Thus, additional sensitization is needed to potentiate the killing effect of TRAIL in these insensitive cancer cells.

Cellular FLICE-inhibitory protein (c-FLIP), a truncated form of caspase-8 that lacks enzymatic activity, is known to be a negative regulator of TRAIL-induced apoptosis by blocking the activation of caspase-8 through competing with caspase-8 for binding to FADD [24]. Numerous studies have documented that elevated c-FLIP expression protects cells from TRAIL-mediated apoptosis, whereas downregulation of c-FLIP by chemicals or small interfering RNA (siRNA) sensitizes cells to TRAIL-induced apoptosis [25]. Elevated levels of c-FLIP have been found in a number of different cancers and are often correlated with a poor prognosis in certain types of cancers [26]. Hence, downregulation of c-FLIP is an effective strategy to sensitize cancer cells to TRAIL-induced apoptosis.

The current study has revealed a previously uncovered biological function of JQ1 and other BET BRD inhibitors in potently decreasing c-FLIP levels and sensitizing cancer cells to TRAIL-induced apoptosis. However these effects are not mediated by inhibition of either BRD4 or c-Myc.

## RESULTS

### JQ1 decreases c-FLIP levels in cancer cell lines

Human non-small cell lung cancer (NSCLC) cell lines display varied sensitivities to JQ1 treatment [6, 14], with some being quite resistant [6]. In an effort to identify agents that suppress c-FLIP expression, we found that JQ1 at a range of 1 to 5 μM effectively decreased the levels of c-FLIP including both long (FLIP_L_) and short (FLIP_S_) forms in the 3 tested JQ1-senstive NSCLC cell lines, H157, H1299 and A549 (Figures. 1B and 1C). Apparent reduction of c-FLIP occurred at 3 h post JQ1 treatment and was sustained up to 16 h (Figure 1D). Under the tested conditions, JQ1 did not obviously increase DR5 expression and decrease the levels of Mcl-1, Bcl-X_L_ and survivin in any of the tested cell lines. JQ1 weakly decreased Bcl-2 levels in H1299 and A549 cells, but not in H157 cells. Interestingly, JQ1 decreased DR4 expression, particularly in H157 and H1299 cells. JQ1 partially decreased c-Myc levels in H1299 cells, but increased its levels in H157 and A549 cells (Figure 1C). Hence, JQ1 effectively decreases c-FLIP levels in cancer cells.

### JQ1 synergizes with TRAIL to induce apoptosis

**Figure 2:**
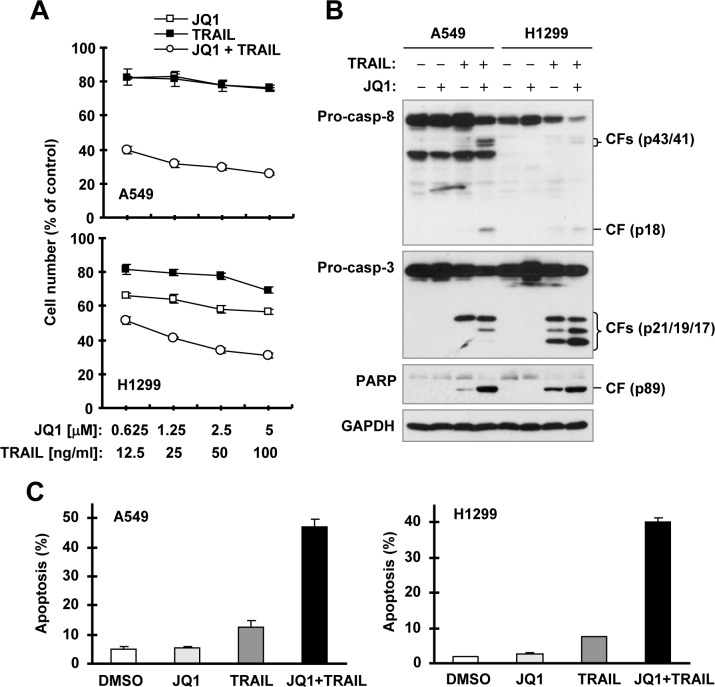
JQ1 synergizes with TRAIL to augment killing of cancer cells and induction of apoptosis (B and C) **A**, The indicated cancer cell lines were treated with different concentrations of JQ1 alone, TRAIL alone, and the combination of JQ1 and TRAIL for 24 h and then subjected to estimation of cell number with the SRB assay. The data are means ± SDs of four replicate determinations. **B** and **C**, The indicated cell lines were treated with 1.25 μM JQ1 alone, 25 ng/ml TRAIL alone or their combination for 14 h and then subjected to preparation of whole-cell protein lysates and subsequent Western blot analysis **(B)** and to detection of apoptosis with annexin V-PE and 7AAD staining followed by flow cytometric analysis **(C)**. CF, cleaved fragment.

Next we determined whether JQ1 sensitizes cancer cells to TRAIL-induced apoptosis due to its c-FLIP-reducing activity. In two TRAIL-insensitive cell lines, A549 and H1299, the combination of JQ1 and TRAIL was much more active than either agent alone in decreasing the survival of cancer cells (Figure 2A). The combination indexes (CIs) for every combination were far less than 1, indicating strong synergy. In agreement, the combination of JQ1 and TRAIL was much more potent than either single agent alone in inducing cleavage of caspase-8, caspase-3 and PARP, hallmarks of apoptosis, and in enhancing Annexin V-positive apoptotic cells in both A549 and H1299 cells (Figures. 2B and 2C). Together these results robustly indicate that the JQ1 and TRAIL combination displays synergistic effect in the induction of apoptosis.

### Enforced ectopic c-FLIP overexpression abolishes synergistic induction of apoptosis by the JQ1 and TRAIL combination

**Figure 3:**
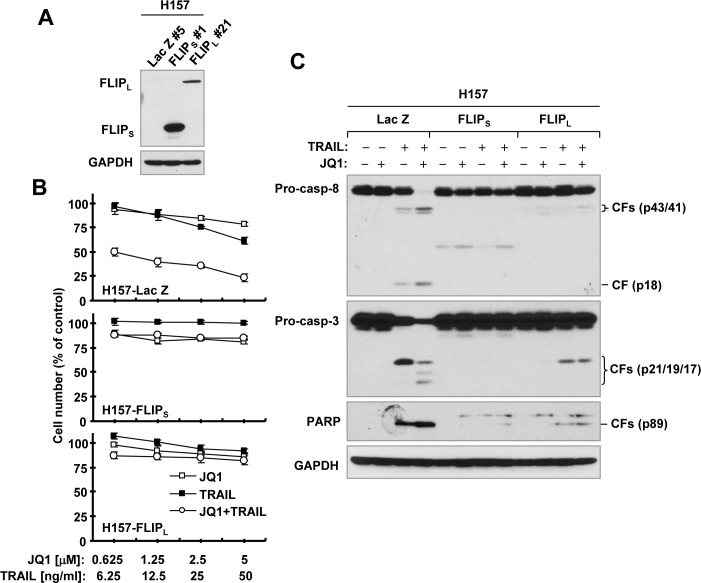
Enforced expression of ectopic c-FLIP (A) abolishes the synergistic effects of JQ1 and TRAIL on decreasing cell survival (B) and activating caspase cascade (C) **A**, Ectopic expression of c-FLIP was verified with Western blotting. **B**, The indicated cell lines were treated with different concentrations of JQ1 alone, TRAIL alone, and the combination of JQ1 and TRAIL for 24 h and then subjected to estimation of cell number with the SRB assay. The data are means ± SDs of four replicate determinations. **C**, The indicated cell lines were treated with 1 μM JQ1 alone, 20 ng/ml TRAIL alone or their combination for 12 h and then subjected to preparation of whole-cell protein lysates and subsequent Western blot analysis. CF, cleaved fragment.

To explore the role of c-FLIP downregulation in the synergistic induction of apoptosis by the JQ1 and TRAIL combination, we compared the effects of JQ1 plus TRAIL on apoptosis induction in H157 cell lines that express ectopic Lac Z (as a control), FLIP_L_ or FLIP_S_ (Figure 3A). Both JQ1 and TRAIL alone minimally or weakly decreased cell survival and induced cleavage of caspase-8, caspase-3 and PARP in H157-Lac Z cells. The combination of JQ1 and TRAIL, however, exerted synergistic effects on decreasing cell survival (CIs < 1) and inducing caspase cleavage in this cell line. These synergistic effects were lost in H157-FLIP_L_ and H157-FLIP_S_ cells (Figures. 3B and 3C). Similar results were also observed in A549 cells that express ectopic FLIP_L_ (data not shown). These data thus clearly suggest that c-FLIP downregulation is critical for the enhancement of TRAIL-induced apoptosis by JQ1.

### JQ1 promotes c-FLIP protein degradation

**Figure 4:**
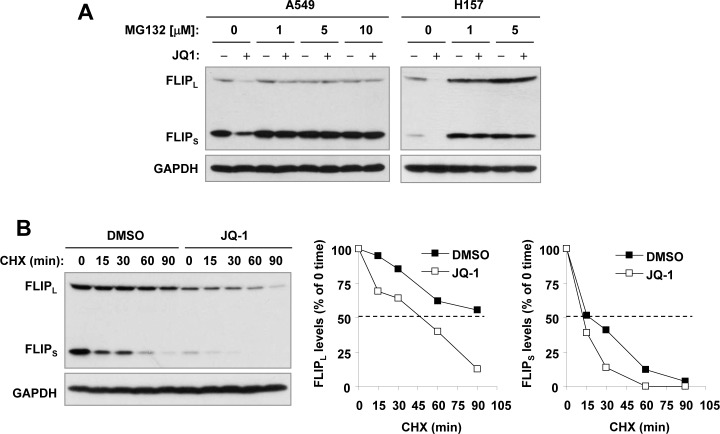
JQ1 decreases levels of c-FLIPs through facilitating their degradation **A**, The indicated cell lines were pretreated with the indicated different concentrations of MG132 for 30 minutes prior to the addition of 1 μM JQ1. After co-treatment for 4 h, the cells were harvested for preparation of whole-cell protein lysates and subsequent Western blot analysis. **B**, A549 cells were treated with DMSO or 1 μM JQ1 for 10 h. The cells were then washed with PBS 3 times and refed with fresh medium containing 10 μg/ml CHX. At the indicated times, the cells were harvested for preparation of whole-cell protein lysates and subsequent Western blot analysis. Protein levels were quantitated with NIH Image J software (Bethesda, MA) and were normalized to actin. The results were plotted as the relative c-FLIP levels compared to those at the time 0 of CHX treatment (right panels).

Given that c-FLIPs are unstable proteins subjected to proteasomal degradation [27, 28], we then determined whether JQ1 facilitates proteasomal degradation of c-FLIP, causing c-FLIP reduction in cancer cells. We found that the presence of the proteasome inhibitor MG132 prevented both forms of c-FLIP from reduction induced by JQ1 (Figure 4A). Moreover, the half-lives of both FLIP_L_ and FLIP_S_ in JQ1-treated cells were shorter than those in DMSO-treated cells (Figure 4B), indicating that JQ1 facilitates c-FLIP degradation or decreases their stability. Collectively these results suggest that JQ1 decreases the levels of c-FLIPs involving facilitation of their degradation.

### JQ1-induced c-FLIP reduction and enhancement of TRAIL-induced apoptosis are independent of BRD4 and c-Myc inhibition

**Figure 5:**
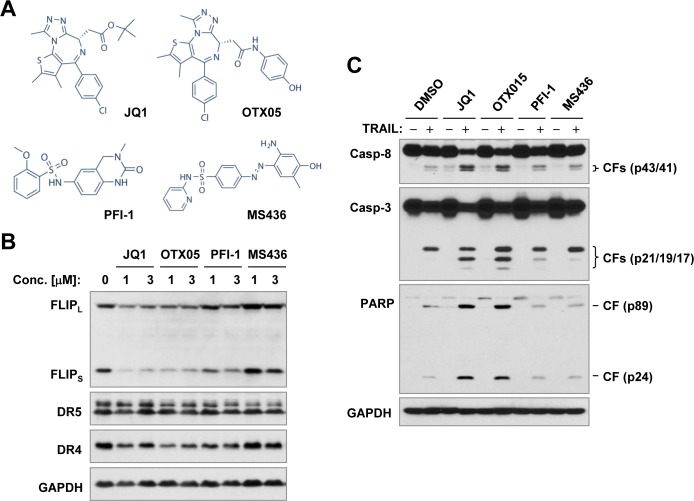
Effects of different BRD inhibitors (A) on decreasing c-FLIP levels (B) and on enhancing TRAIL-induced apoptosis (C) **A**, Chemical structures of the tested BRD inhibitors. **B** and **C**, A549 cells were exposed to the different concentrations of inhibitors as indicated for 8 h **(B)** or treated with 1 μM of a given BRD inhibitor alone, 50 ng/ml TRAIL alone or their respective combinations for 14 h **(C)**. The cells were then harvested for preparation of whole-cell protein lysates and subsequent Western blot analysis for detection of the indicated proteins. CF, cleaved fragment.

One putative mechanism of action of BET BRD inhibitors involves suppression of c-Myc [3, 5]. To determine whether JQ1-induced c-FLIP reduction is the consequence of BRD4 or c-Myc inhibition, we first analyzed the effects of other BET BRD inhibitors on altering c-FLIP levels and enhancing TRAIL-induced apoptosis. OTX015, a compound highly related to JQ1 in chemical structure (Figure 5A), was as effective as JQ1 in decreasing the levels of c-FLIP (Figure 5B). PFI-1 and MS436, which have distinct chemical structures from JQ1, either weakly decreased (PFI-1) or even slightly increased (MS436) c-FLIP levels (Figure 5B). Consistently, OTX05, but not PFI-1 and MS436, enhanced TRAIL-induced apoptosis, as evidenced by increased cleavage of caspase-8, caspase-3 and PARP, as did JQ1 (Figure 5C).

**Figure 6:**
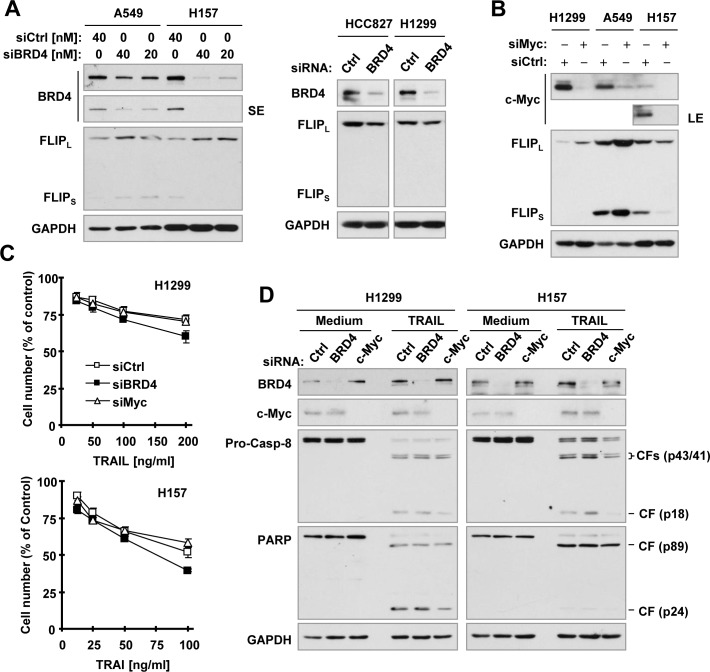
Effects of siRNA-mediated genetic suppression of BRD4 or c-Myc on modulating c-FLIP levels (A and B) and TRAIL-induced apoptosis (C and D) **A** and **B**, The indicated cancer cell lines were transfected with control (Ctrl), BRD4 or c-Myc siRNA for 48 h and then harvested for preparation of whole-cell protein lysates and subsequent Western blot analysis. **C** and **D**, The indicated cell lines were transfected with control (Ctrl), BRD4 or c-Myc siRNA for 24 h and re-plated in 96-well plates and 6-well plates. On the second day, the cells in 96-well plates **(C)** were treated with different concentrations of TRAIL for 24 h and then subjected to estimation of cell number with the SRB assay. The data are means ± SDs of four replicate determinations. The cells in 6-well plates **(D)** were treated with 100 ng/ml (H1299) or 50 ng/ml (H157) TRAIL for 24 h and then subjected to preparation of whole-cell protein lysates and subsequent Western blot analysis. CF, cleaved fragment.

Moreover, we used a more specific genetic approach by directly knocking down BRD4 or c-Myc and investigating the impact of their knockdown on c-FLIP levels and TRAIL-induced apoptosis. Knockdown of BRD4 actually slightly increased c-FLIP levels in A549 and H157 cells, but slightly decreased c-FLIP levels in H1299 and HCC827 cells (Figure. 6A). Similarly, knockdown of c-Myc increased c-FLIP levels in H1299 and A549 cells, but decreased c-FLIP levels in H157 cells (Figure 6B). Hence it is clear that knockdown of both BRD4 and c-Myc generates mixed effects on c-FLIP levels depending on cell lines. Moreover, knockdown of either BRD4 or c-Myc did not apparently enhance the potency of TRAIL in decreasing cell survival (Figure 6C) and in inducing cleavage of caspase-8, caspase-3 and PARP (Figure 6D) in both H1299 and A549 cells, indicating that genetic suppression of BRD4 or c-Myc did not accordingly sensitize cancer cells to TRAIL-induced apoptosis. Taking these data together, we suggest that genetic inhibition of BRD4 or c-Myc does not mimic the ability of JQ1 to decrease c-FLIP levels and enhance TRAIL-induced apoptosis.

### Inhibition of c-Myc elevation fails to abolish the ability of JQ1 to reduce c-FLIP levels

**Figure 7:**
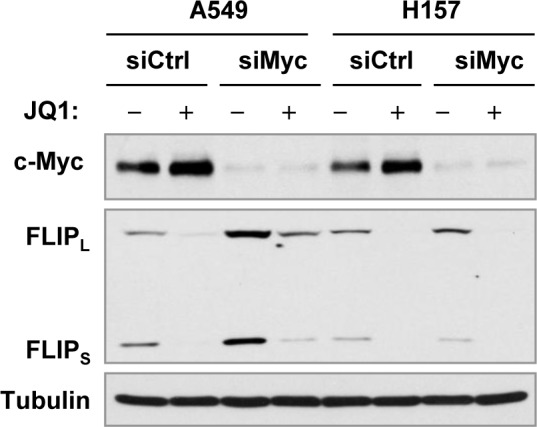
Effects of c-Myc knockdown on JQ1-induced c-FLIP reduction in cancer cell lines in which c-Myc expression is increased by JQ1 treatment The indicated cell lines were transfected with control (Ctrl) or c-Myc siRNA (siMyc) for 38 h and then exposed to DMSO or 2 μM JQ1 for additional 12 h. The cells were then harvested for preparation of whole-cell protein lysates and subsequent Western blot analysis.

In our study, we found that JQ1 increased the levels of c-Myc in A549 and H157 cells as presented in Figure 1. A previous study has suggested that c-Myc directly suppresses c-FLIP gene expression [29]. To determine whether JQ1-induced c-FLIP reduction is connected to c-Myc upregulation in these two cell lines, we used c-Myc siRNA transfection to knock down c-Myc expression including c-Myc upregulation induced by JQ1 and then examined its impact on c-FLIP reduction induced by JQ1. We found that c-Myc siRNA effectively decreased c-Myc expression, but failed to prevent c-FLIP reduction induced by JQ1 although the basal levels of c-FLIP were elevated by c-Myc knockdown in A549 cells (Figure 7), suggesting that JQ1-induced c-FLIP reduction is unlikely secondary to c-Myc upregulation in these cell lines.

## DISCUSSION

The current study has shown that JQ1 decreases c-FLIP levels and enhances TRAIL-induced apoptosis in cancer cells, uncovering a novel biological function of JQ1 in the regulation of the extrinsic apoptotic pathway. In this study, JQ1 substantially decreased c-FLIP (both FLIP_L_ and FLIP_S_) levels without increasing the expression of DR4 and DR5, two other critical components in mediating TRAIL-induced apoptosis [22]. Moreover, enforced expression of ectopic c-FLIP abrogates the ability of JQ1 to enhance TRAIL-induced apoptosis. Therefore, it is very likely that JQ1 synergizes with TRAIL to augment apoptosis primarily through c-FLIP downregulation. Although suppression of other anti-apoptotic proteins such as Bcl-2 family members and survivin sensitizes cancer cells to TRAIL-induced apoptosis [30], JQ1 did not decrease the levels of Bcl-X_L_, Mcl-1 and survivin in every tested cell lines except for weak effects on reducing Bcl-2 in H1299 and A549 cells. Therefore, JQ1-induced enhancement of TRAIL-induced apoptosis is not associated with modulation of these proteins.

Protein ubiquitinylation and proteasomal degradation is a key mechanism that regulates c-FLIP levels [25, 31]. In this study, inhibition of the proteasome with MG132 rescued c-FLIP downregulation caused by JQ1. Moreover JQ1 treatment destabilized c-FLIP proteins by facilitating their degradation. Hence it is clear that JQ1 decreases c-FLIP levels by promoting their proteasomal degradation.

The majority of studies to date have shown that the therapeutic effect of BET BRD inhibitors is largely due to targeting BRD4, rather than the other BET proteins [2]. Since JQ1 is primarily a BRD4 inhibitor [4], it is reasonable to ask whether inhibition of BRD4 accounts for c-FLIP reduction induced by JQ1. Further study with additional BET BRD inhibitors including OTX015, PFI-1 and MS436 found that OTX015, but not PFI-1 and MS436, displayed similar effects as JQ1 did in decreasing c-FLIP levels and enhancing TRAIL-induced apoptosis. While PFI-1 and MS436 are selective BRD4 inhibitors [9, 32], OTX015 is an inhibitor of BRD2, BRD3 and BRD4 [33]. However, OTX015 is chemically closer to JQ1 because they share similar parental chemical structures, while PFI-1 and MS436 have relatively distinct chemical structures (Figure 6A). Interestingly, it is OTX015, but not PFI-1 and MS432, that functions as effectively as JQ1 in decreasing c-FLIP levels and enhancing TRAIL-induced apoptosis. It seems that JQ1 and OTX015 share similar activity in modulating c-FLIP levels due to their chemical similarity, rather than inhibition of BRD4. Suppression of BRD4 with siRNA-mediated gene knockdown did not result in substantial reduction of c-FLIP and enhancement of TRAIL-induced apoptosis. Instead, we observed an increase in c-FLIP in some cell lines. Hence, the genetic suppression of BRD4 fails to mimic the effect of JQ1 in decreasing c-FLIP and sensitizing cancer cells to TRAIL-induced apoptosis. Collectively, we conclude that JQ1 decreases c-FLIP levels and enhances TRAIL-induced apoptosis independent of BRD4 inhibition.

While c-Myc has been suggested to be a putative target gene that mediates the cancer therapeutic activity of JQ1 and other BET BRD inhibitors [2, 3, 5], increased recent studies have suggested that these inhibitors exert c-Myc-independent activity [2, 6, 7]. In our study, JQ1 in fact increased c-Myc levels in H157 and A549 cells but decreased its levels in H1299 cells. This finding is in agreement with a previous study that shows c-Myc upregulation by JQ1 in some lung cancer cell lines [6]. Genetic suppression of c-Myc through siRNA-mediated gene knockdown did not substantially decrease c-FLIP levels, rather increased c-FLIP levels in some cancer cell lines (e.g., A549 and H1299). Moreover we did not find that genetic suppression of c-Myc mimicked the ability of JQ1 to enhance TRAIL-induced apoptosis. Therefore it is unlikely that JQ1 decreases c-FLIP levels and enhances TRAIL-induced apoptosis through c-Myc suppression.

A previous study has suggested that c-Myc suppresses c-FLIP by directly repressing its transcription [29]. In agreement, we found that c-Myc knockdown indeed elevated basal levels of c-FLIP in some cell lines (e.g., A549 and H1299). The c-Myc elevation in some cell lines (e.g., A549 and H157) exposed to JQ1 raised the possibility that c-FLIP reduction by JQ1 is secondary to c-Myc upregualtion, at least in these cell lines. However, suppression of c-Myc upregulation by knocking down its expression failed to affect the ability of JQ1 to decrease c-FLIP levels in both A549 and H157 cells although it elevated basal levels of c-FLIP in A549 cells. Combining with the data that JQ1 decreased the levels of both c-FLIP and c-Myc in H1299 cells, we suggest that c-FLIP reduction induced by JQ1 is unlikely due to c-Myc upregulation either.

Hence, the mechanism by which JQ1 decrease c-FLIP levels via facilitating their degradation has not been elucidated in this study and thus deserves further investigation in the future. The general mechanisms accounting for degradation of both FLIP_L_ and FLIP_S_ have not been fully elucidated by now. Our findings in this study suggest that JQ1 can be a valuable research tool for understanding c-FLIP degradation mechanisms.

Targeting the TRAIL/death receptor signaling pathway with either recombinant TRAIL or agonistic death receptor antibodies has been considered a promising cancer therapeutic strategy and has been tested in the clinic [20-23]. Our findings warrant further investigation of JQ1 or OTX015 as a sensitizer of TRAIL/DR-targeted cancer therapy *in vivo* and in the clinic, thus promising the clinical translational significance of this approach.

## MATERIALS AND METHODS

### Reagents

(+)-JQ1 was purchased from ApexBio (Houston, TX). OTX015 and MS436 were purchased from Cayman Chemical (Ann Arbor, MI). Soluble recombinant human TRAIL was purchased from PeproTech, Inc. (Rocky Hill, NJ). MG132, cycloheximide (CHX) and PFI-1 were purchased from Sigma Chemical Co. (St. Louis, MO). Monoclonal anti-FLIP antibody (NF6) was obtained from Alexis Biochemicals (San Diego, CA). Mouse monoclonal caspase-8, PARP, survivin and c-Myc antibodies were purchased from Cell Signaling Technology, Inc. (Danvers, MA). Mouse monoclonal caspase-3 antibody was purchased from Imgenex (San Diego, CA). Rabbit polyclonal DR5 antibody was obtained from ProSci Inc. (Poway, CA). Mouse monoclonal DR4 antibody (B-N28) was purchased from Diaclone (Stamford, CT). Rabbit polyclonal Mcl-1 and Bcl-X_L/S_ and mouse monoclonal Bcl-2 antibody were purchased from Santa Cruz Biotechnology, Inc. (Santa Cruz, CA). Tubulin and GAPDH antibodies were purchased from Sigma Chemical Co. and Trevigen Inc. (Gaithersburg, MD), respectively.

### Cell lines and cell culture

Human cancer cell lines used in this study were described in our previous work [34]. Except for H157 and A549 cells, which were authenticated by Genetica DNA Laboratories, Inc. (Cincinnati, OH) through analyzing short tandem repeat DNA profile, other cell lines have not been authenticated. The stable cell lines, H157-Lac Z #5, H157-FLIP_S_ #1 and H157-FLIP_L_ #21 were described previously [35, 36]. A549-Lac Z #2, A549-Lac Z #9 and A549-FLIP_L_ #4 were described in our previous studies [37, 38]. These cell lines were cultured in RPMI 1640 medium containing 5% fetal bovine serum at 37°C in a humidified atmosphere of 5% CO_2_ and 95% air.

### Cell survival and apoptosis assays

Cells were seeded in 96-well cell culture plates and treated the next day with the given agents. Viable cell numbers were determined using sulforhodamine B (SRB) assay as described previously [39]. CI for drug interaction (e.g., synergy) was calculated using the CompuSyn software (ComboSyn, Inc.; Paramus, NJ). Apoptosis was evaluated by detection of caspase and PARP cleavage with Western blot analysis and with a PE Annexin V Apoptosis Detection kit (BD Biosciences; San Jose, CA) according to the manufacturer's instructions.

### Western blot analysis

Preparation of whole-cell protein lysates and performance of the Western blot analysis were the same as described previously [40].

### Gene knockdown by siRNA

BRD4 (sc-43639) and c-Myc (#6341) siRNAs were purchased from Santa Cruz Biotechnology (Santa Cruz, CA) and Cell Signaling Technology, Inc., respectively. Transfection of these siRNA duplexes was conducted in 6-well plates using the HiPerFect transfection reagent (Qiagen) following the manufacturer's manual.
